# Radiobiological effects and proton RBE determined by wildtype zebrafish embryos

**DOI:** 10.1371/journal.pone.0206879

**Published:** 2018-11-08

**Authors:** Emília Rita Szabó, Michael Brand, Stefan Hans, Katalin Hideghéty, Leonhard Karsch, Elisabeth Lessmann, Jörg Pawelke, Michael Schürer, Elke Beyreuther

**Affiliations:** 1 Attosecond Light Pulse Source, ELI-HU Nonprofit Ltd., Szeged, Hungary; 2 Center for Molecular and Cellular Bioengeneering (CMCB), DFG-Center for Regenerative Therapies Dresden (CRTD), Technische Universität Dresden, Dresden, Germany; 3 Helmholtz-Zentrum Dresden – Rossendorf, Dresden, Germany; 4 OncoRay – National Center for Radiation Research in Oncology, Faculty of Medicine and University Hospital Carl Gustav Carus, Technische Universität Dresden, Helmholtz-Zentrum Dresden - Rossendorf, Dresden, Germany; 5 National Center for Tumor Diseases (NCT), partner site Dresden, Germany; North Shore Long Island Jewish Health System, UNITED STATES

## Abstract

The increasing use of proton radiotherapy during the last decade and the rising number of long-term survivors has given rise to a vital discussion on potential effects on normal tissue. So far, deviations from clinically applied generic RBE (relative biological effectiveness) of 1.1 were only obtained by *in vitro* studies, whereas indications from *in vivo* trials and clinical studies are rare. In the present work, wildtype zebrafish embryos (*Danio rerio*) were used to characterize the effects of plateau and mid-SOBP (spread-out Bragg peak) proton radiation relative to that induced by clinical MV photon beam reference. Based on embryonic survival data, RBE values of 1.13 ± 0.08 and of 1.20 ± 0.04 were determined four days after irradiations with 20 Gy plateau and SOBP protons relative to 6 MV photon beams. These RBE values were confirmed by relating the rates of embryos with morphological abnormalities for the respective radiation qualities and doses. Besides survival, the rate of spine bending, as one type of developmental abnormality, and of pericardial edema, as an example for acute radiation effects, were assessed. The results revealed that independent on radiation quality both rates increased with time approaching almost 100% at the 4^th^ day post irradiation with doses higher than 15 Gy. To sum up, the applicability of the zebrafish embryo as a robust and simple alternative model for *in vivo* characterization of radiobiological effects in normal tissue was validated and the obtained RBE values are comparable to previous finding in animal trials.

## Introduction

Proton radiotherapy (PT) has rapidly evolved from the pioneering trials at the end of the 20^th^ century to become an accepted alternative to conventional external beam radiotherapy (EBRT) with photons. The superior physical depth dose distribution with higher dose deposition at the end of the proton track, in the Bragg peak, means that PT offers the possibility of sufficient dose delivery to the tumor whilst simultaneously sparing the surrounding normal tissue. By contrast, larger volumes of normal tissues are still affected by MV photon EBRT techniques, even with advanced techniques like intensity-modulated radiotherapy.

During the last years, the more widespread application and the increasing numbers of patients and long-time survivors treated with PT give rise to discussions on the biological response to proton radiation [1]. So far, treatment planning and evaluation in PT is based on a fixed generic relative biological effectiveness (RBE) of 1.1, which describes a 10% higher cell killing efficiency for protons relative to photons. Contrary to the clinical practice [2] of using a constant factor experimental evidence indicates a variable RBE with increasing values at the end of the proton range, where the remaining low energetic protons deposit a larger amount of energy per path length (as expressed in the term of linear energy transfer (LET)). This increased LET is correlated to an enhanced RBE which might be of risk for the surrounding normal tissue in particular in the normal tissue distal of the tumor [1,3]. One of the few clinical studies that provide evidence for such variable RBE was published by Peeler et al. [4] who correlate alterations in magnetic resonance images with RBE variations of clinical proton depth dose curves.

Several *in vitro* experiments have been performed in order to resolve the RBE-LET dependency with higher spatial resolution, which revealed RBE values between 1.1–1.2 in the middle of a spread-out Bragg peak (SOBP) and 1.6 at the distal end of the depth dose distribution [3,5,6]. However, verification of these values by dedicated *in vivo* trials is still pending. The limited available animal data either confirms the fixed RBE of 1.1 or reveals a less pronounced increase than the value of 1.6 found in *in vitro* cell studies [3,7–10]. Exemplarily, moderately increased RBE values of 1.13 ± 0.04 for the entrance region and of 1.26 ± 0.05 at the distal end of a 6 cm proton SOBP were found [9] rating the occurrence of myelopathies after rat spinal cord irradiation and using MV photons as reference. One of the main challenges for such purposive *in vivo* studies on the RBE-LET correlation is the precise and reproducible positioning of animals and target volumes along the proton depth dose distribution. Likewise in clinical patient treatment anatomical changes, positioning uncertainties and organ movements might result in deviations from the desired beam position in the body, especially at the distal end of the proton path (i.e. the proton range). These unavoidable uncertainties and variations for individual patients and in between fractions were considered in patient treatment by means of mm-wide safety margins around the tumor volume, whereas for animal studies they result in a smearing of the distal high-LET effect. *In vivo* proton range verification [11] and more sophisticated imaging techniques, like proton radiography [12], are some of the possibilities that might help to meet the requirements on beam positioning accuracy. Another way to achieve smaller proton range uncertainties is the application of animal models that are much smaller and anatomical less inhomogeneous (no air and bone) than the standard rodents.

In the present work, zebrafish embryos are used as an alternative *in vivo* model to resolve differences in the biological response to proton radiation. With an irradiation size of about 1 mm, between cell monolayer culture and subcutaneous tumors or normal tissue organs in small animals (mice, rat), the zebrafish embryo could potentially be deployed for detailed investigations on the RBE-LET correlation. Sampling is conceivable for each mm of the proton depth dose distribution, especially at the distal edge of the SOBP. This combined with other properties, including a high genetic similarity of 70% to human, e.g., genes of the DNA repair machinery [13], a high number of progeny, their rapid development and optical transparency that facilitate continuous observation of organ perturbations favors the zebrafish embryo for radiation research of normal tissue response [14–18].

The zebrafish embryo as a small vertebrate model for comparative radiobiology studies with protons was established in the present work as a first step towards detailed mm-wise investigations on the biological effects along the proton path. Therefore, zebrafish embryos were irradiated at two different depth positions, i.e. at the entrance plateau and in the middle of the SOBP (hereafter named mid-SOBP), using a 150 MeV proton beam of the University Proton Therapy Dresden (UPTD). The dose dependent embryonic survival and induction of pericardial edema and malformations were recorded within the first days post treatment and compared to those induced by the 6 MV clinical photon reference.

## Material and methods

### Zebrafish embryo handling and irradiation preparation

The experiments were implemented using laboratory bred wild type (AB) strain of zebrafish (*Dario rerio*) embryos. Animal experimental protocol was planned according to the European Parliament and Council (EU Directive 2010/63/EU) on the protection of animals used for scientific purposes, which comprises that the early life-stage of zebrafish are not protected and thus no ethical approval is required until the stage of being capable of independent feeding (5 days post fertilization). All procedures were performed with respect to this directive and in accordance with German legislation on the care and use of laboratory animals.

Zebrafish embryos were kindly provided by the Center for Regenerative Therapies at Technische Universität Dresden. Embryos were washed and sorted into E3 medium (5 mM NaCl, 0.17 mM KCl, 0.33 mM CaCl2, 0.33 mM MgSO4, 0.1% methylene blue [19]), afterwards transported for irradiation at 21–22 hour post fertilization (hpf) period with care of the necessary temperature maintenance. For this study, embryos at 24 hpf, i.e. in pharyngula period (24–48 hpf) were applied as easily ascertainable by pigmentation of the retina [18]. The embryos were maintained at different temperatures to slow normal embryogenesis [20] in order to ensure the synchronization of developmental stages of the collected embryos and to compensate for different irradiation time points at the two clinical accelerators, (morning for protons, evening for photons). Embryos scheduled for proton treatment were kept at normal room temperature (22–24 °C) from 22 hpf on whilst those scheduled for photon irradiation were first maintained at 22 °C overnight to slow down development and subsequently kept at normal temperature until irradiation. The developmental stage (24 hpf) of the embryos was checked immediately before irradiation by microscopic observation (Axiovert S100, Zeiss, Jena, Germany) and after irradiation the embryos were maintained at 28 °C. The time point of final assessment was maximal 95 hours or almost 4 days post irradiation (dpi), which is equivalent to 119 hpf. The embryos were then sacrificed and fixed in 4% paraformaldehyde for subsequent histological analysis.

**Fig 1:**
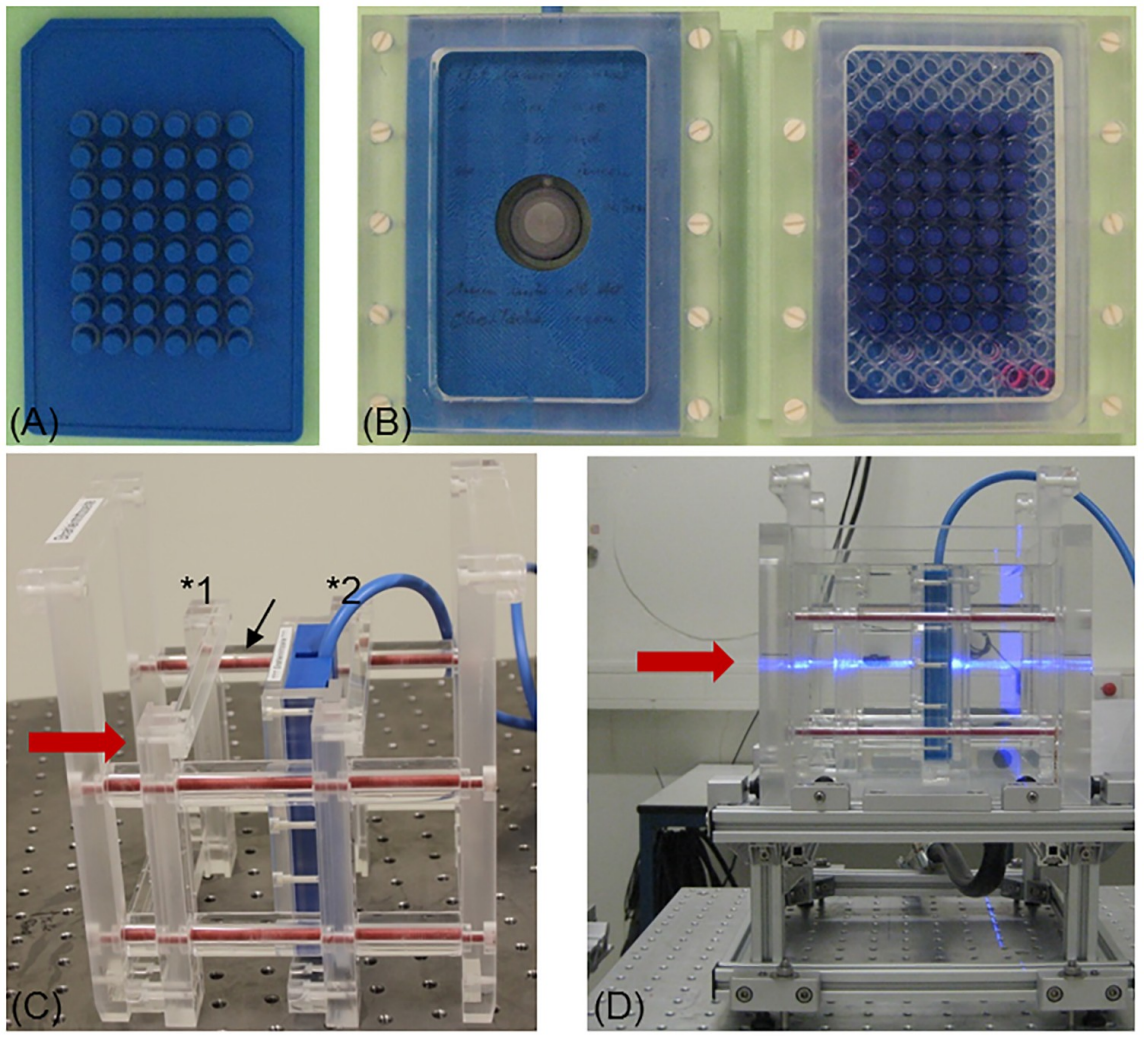
Setup and equipment for zebrafish embryo irradiation at horizontal beams. (A) ABS stamp and (B) exemplary sample holder for the capped Markus IC (left) and one 96 well plate covered by a stamp plate (right) with the inner 48 wells filled with embryo medium. (C) Water phantom insert with holder and Markus IC at mid-SOBP position (*2). The second, empty holder is fixed by PMMA spacers (arrow) at entrance plateau (*1) position. (D) The complete water filled phantom at the horizontal proton beam (coming from left and marked by the horizontal laser line in light blue) with holder (dark blue) including Markus IC in upright position at mid-SOBP.

For irradiation, two zebrafish embryos in 200 μl E3 were placed in each of the inner 48 wells of 96 well plates (Corning via Sigma-Aldrich Chemie GmbH, Munich, Germany) with the upper and lower row and the two outer columns blank. Transportation and storage of non-treated samples occurred in warmed polystyrene boxes and control plates were treated in a similar way, except irradiation. The 96 well plates have to be irradiated upright due to the horizontal beam delivery with bottom side of the plate (polystyrene plastic of 1.1 mm thickness) facing the beam. Medium leakage was prevented by stamps that match the reverse profile of the inner 48 wells (Fig 1A and 1B). The corresponding reduction in well volume to 3 mm in height did not interfere with proper embryonic development, i.e. there was no significant reduction in embryo survival due to the experiment conditions observed.

### Setup for reproducible irradiation at horizontal beams

A water filled cell phantom is available at UPTD (Fig 1C and 1D) for the reproducible and precise positioning and irradiation of zebrafish embryos in 96 well plates. The phantom and a comprehensive description of its dosimetric characterization are given elsewhere [21]. Briefly, the phantom consists of PMMA (polymethyl-methacrylate) walls that surround a water volume of 180.0 x 180.0 x 171.9 mm³ (width x height x length, i.e. horizontal lateral extension x vertical lateral extension x depth along the beam axis). The PMMA wall has a defined thickness of 12.00 ± 0.05 mm for the side where the beam enters and the samples can be positioned along beam axis by moving their holders along four polyvinylchloride bars in parallel to beam axis. The positions of holder and samples are fixed by PMMA cylinders of appropriate size, which results in a depth positioning accuracy of ± 0.2 mm. Likewise, the usage of multiple holders and PMMA cylinders allow for reproducible and fast positioning of samples at different depths. In the present experiment two positions were set in such a way that the embryos were irradiated with protons of the entrance plateau or in mid-SOBP position. The mid-SOBP position was also applied for photon irradiation. A compilation of the different geometrical distances, the conversion factors and resulting water equivalent path lengths (WEPL), resp. the positions of the zebrafish embryos in depth, for the different radiation qualities are given in Table 1. The position of the embryos was generally assumed to be halfway through the available space (3 mm height), when the 96 well plates were turned upright.

**Table 1:** Geometrical and dosimetric path lengths of materials in the beam path.

Material in beam path	Protons			6 MV Photons	
	Conv. factor	WEPL /mm		Conv. factor	WEPL /mm
		Plateau	Mid-SOBP		
Cell phantom entrance window, 12.0 mm PMMA	1.163	13.96		1.136	13.63
Plastic bottom of 96 well plate, 1.1 mm polystyrene	1.023	1.12		1.0	1.1
Water	1.0	13.9	88.4	1.0	88.4
Embryo medium^a^, 1.5 mm water	1.0	1.5		1.0	1.5
Position of zebrafish embryo in depth of water		30.5	105.0		104.6

Overview of geometrical and dosimetric parameters of the different materials in the beam path from beam entrance into the cell phantom through to the respective positions of the zebrafish embryos. The corresponding conversion factors (stopping power ratios, [21]) are given for completeness.

^a^Embryos were considered as water equivalent and positioned halfway through the 3 mm available space.

For irradiation, the 96 well plates were transported enclosed with their normal lids, which were replaced by stamps directly before irradiation. The 96 well plate were then enclosed with the stamp and inserted in one holder (Fig 1B) and at the corresponding position within the irradiation phantom (Fig 1C) facing the plate bottom towards the beam.

### Irradiation with 150 MeV protons at UPTD

In the experimental hall of the UPTD a fixed horizontal beamline is installed that deliver monoenergetic proton beams in the energy range of 70–230 MeV, whereas in the present study only 150 MeV protons were applied. For 150 MeV protons, a beam shaping system consisting of a double-scattering device and a ridge filter provides a laterally extended proton field of 10 x 10 cm^2^ size and a SOBP of variable widths between 20–32 mm in water [22]. For the present experiments, a SOBP with a modulation width of 26.3 mm (90% dose plateau) was applied ranging from 86.0 mm to 112.3 mm depth in water. The lateral dose homogeneity is comparable to that used for patient treatment with relative dose variations of less than ± 2% over the extended proton field.

The daily quality assurance routine includes the verification of the lateral dose homogeneity at both positions of the proton depth dose curve. Therefore, the phantom is removed and the 2D dose distribution of the irradiation field is depicted with a Lynx scintillation detector (IBA Dosimetry GmbH, Schwarzenbruck, Germany) revealing potential influences on the irradiation field arising from positional changes of the beam shaping system. Thereby, the depth positions of entrance plateau and mid-SOBP were adjusted by one and eleven 7.7 mm thick polycarbonate (PC) plates (water equivalent path length = 1.15 times PC thickness) in front of the Lynx detector, respectively. The dose homogeneity over the irradiation field inside the cell phantom was additionally proven by GafChromic EBT3 dosimetry films (ISP Corp., New York, USA) that were placed directly behind a 96 well plate. After irradiation, the films were air dried, scanned at least two days after irradiation with an Epson Expression Flatbed Scanner (Epson, Meerbusch, Germany) and analyzed by IDL (Interactive Data Language, Harris Geospatial Solutions, Exelis Visual Information Solutions GmbH, Gilching, Germany) based software [23]. The resulting dose distribution confirms lateral field inhomogeneity less than ± 3%. Exemplarily, an EBT3 film used to assess the dose homogeneity at the entrance plateau is shown in S1 Fig.

The positioning at entrance plateau and mid-SOBP within the cell phantom was additionally checked by a capped Markus ionization chamber (IC; model 34045, PTW, Freiburg, Germany; 1.06 mm water equivalent thickness of the entrance window) at sample position. For this, the Markus IC is placed into a special holder (Fig 1B) that in turn can be inserted into the holder for the 96 well plates and assures equal positioning of measuring volume and sample. By shifting the proton depth dose curve with PC plates in front of the cell phantom and dose measurements with Markus IC the distance of the sample positions to the distal edge of the SOBP was validated. Moreover, the relative depth dose distribution and therefore sample positioning, was also confirmed by means of EBT3 film stacks.

Similar to radiotherapy, the dose delivery at the experimental proton beam line is monitored by a beam transmission IC (model 34058, PTW) at beam exit that switches off the beam after reaching the requested number of monitor units (MU). Correlations between the radiated MU and absolute dose to water at sample position were determined as part of the daily routine by measurements with the Markus IC at sample position. There, a constant dose rate of 5 Gy/min was set at both sample depth positions, whereas a higher beam current has to be applied for irradiation in the plateau region to compensate for differences in the dose deposition at the two positions. A maximum uncertainty of 4.7% was estimated for the absolute dose to water delivered to the zebrafish embryos which considers the uncertainties of air temperature and pressure during dose measurement, IC calibration and beam quality correction factors. The correlation between radiated MU and absolute dose at sample position was confirmed at the end of each experiment day and in between sample irradiation.

### Photon reference irradiation

Photon reference irradiations were performed at a clinical linac type Artiste (Siemens, Erlangen, Germany) on-site at UPTD that was rotated for horizontal delivery of 6 MV photon beams comparable to the proton irradiation. Unlike for the proton experiments, the necessary dosimetry for radiobiological experiments at the linac could be related to clinical standard dosimetry with an IC10 (Wellhöfer/IBA Dosimetry) in a homogeneous water filled BluePhantom (IBA Dosimetry).

Similar to the proton treatment, zebrafish embryos were irradiated in 96 well plates and, for practical reasons, also in the same position as for mid-SOBP (see Table 1 for details). Correlations between radiated MU and absolute dose to water at sample position were determined based on clinical standard dosimetry, i.e. correcting the relation of 100 MU = 1 Gy for deviations from standard reference field conditions by using clinical output factors. In addition, the absolute dose at sample position was measured with a semiflex IC (model 31010, PTW). Analogue to the Markus IC for proton dosimetry, a special holder was made replacing the holder for the 96 well plates and ensuring that the measuring volume of the semiflex IC was at the same position as the zebrafish embryos. At this position, a dose rate of 2.86 Gy/min was achieved. For this approach, the maximum dose uncertainty was estimated by 3.7% including the uncertainty of clinical daily dosimetry QA as well as the uncertainties arising from the deviation of air temperature and pressure during dose measurement, and depth position uncertainty of the embryos within the sample wells. Again, the photon lateral field homogeneity at sample position was confirmed by EBT3 films behind a 96 well plate, showing dose homogeneity better than 97% over the irradiated area.

### Survival and morphology analysis

**Fig 2:**
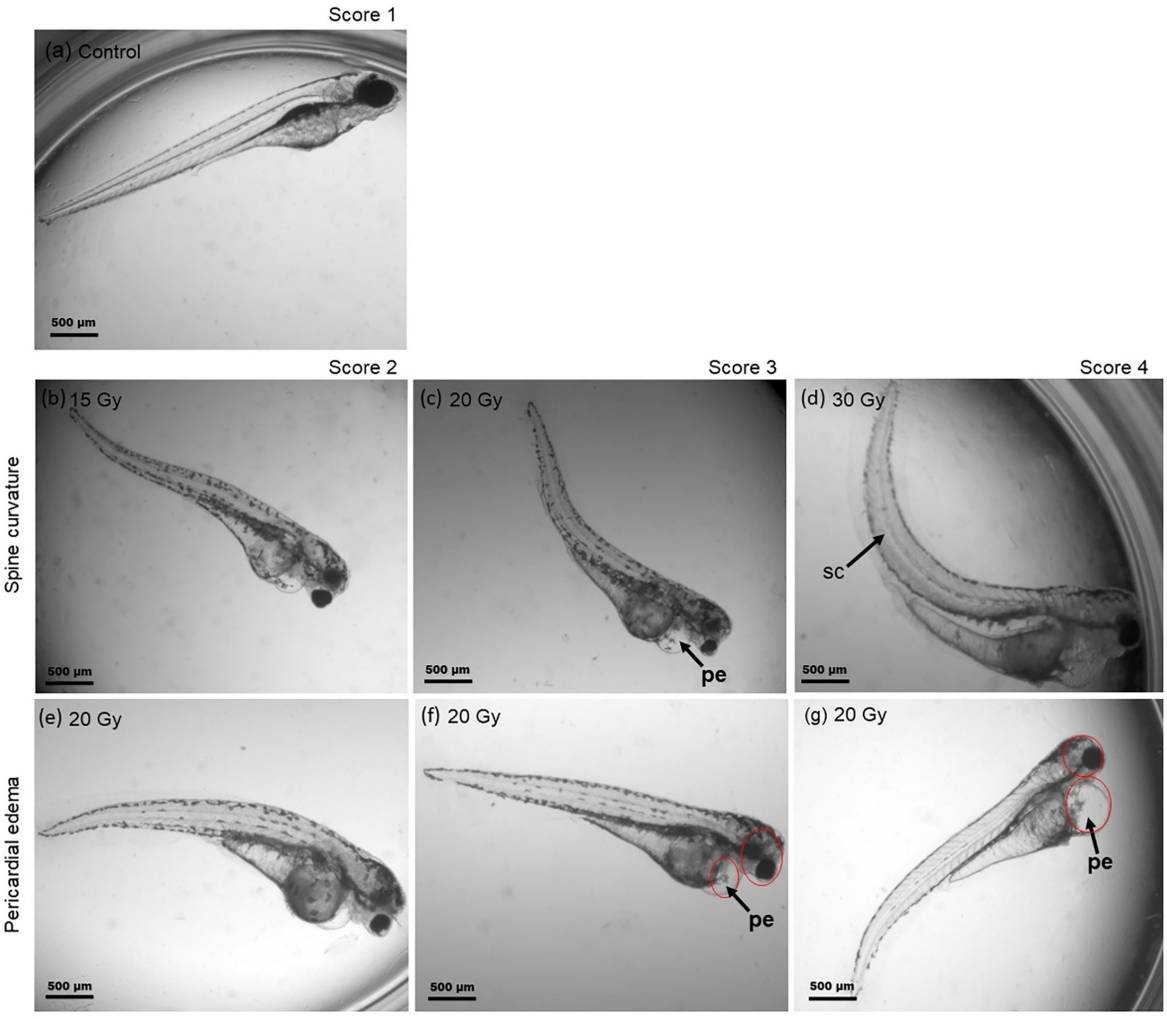
Scoring system for radiation induced morphological malformations. (a) Normal developed zebrafish embryo at 4 dpi and (b-g) representative examples of malformations observed at 4 dpi with protons and photons during pharyngula period. Spine bending with increasing severity (b-d) is shown in the 2^nd^ row, whereas the last row shows the corresponding scoring for the induced pericardial edema (e-g).

The viability of the embryos was immediately assessed after irradiation using an inverted microscope (Axiovert S100, Zeiss, Jena, Germany). Subsequently, embryos were separated in one embryo per well and maintained under normal conditions (28.0 °C, 14 h light/10 h dark cycle) with an exchange of embryo medium every second day. The developmental status of the embryos, hatching rate, survival and morphological abnormalities, i.e., spine curvature (Fig 2b–2d) and pericardial edema (Fig 2e–2g), were monitored daily to the end of the observation time with a Zeiss Observer Z1 microscope (magnification: 50x). On the 3^rd^ (72 hpf) and 4^th^ (119 hpf) day of observation representative images were recorded from the surviving and malformed embryos for quantitative morphological assessment using an AxioCam MRm at a Zeiss Axiovert 40 CFL microscope under a magnification of 50x. The embryonic survival was defined through the assessment of heartbeat and blood circulation.

### Scoring system for quantitative analysis of embryo malformations

A morphological scoring system developed by Brannen et al. [24] on basis of measured morphological abnormalities was adapted in order to categorize the observed pericardial edema and spinal curvature. Pictures of individual embryos at 3 dpi and 4 dpi were retrospectively assessed and the visible malformations categorized in accordance to the scoring system depicted in Fig 2. In the numerical scoring system for the spinal curvature the score of 1 represented a normal spine (2a), the score of 2 a curved end of tail (2b), the score of 3 a slight bending from half of the body (2c), and the score of 4 the most severe curvature (2d). For pericardial edema, the corresponding score values represent score 1 the normal healthy state of the embryos (2a), score 2 a variation within the normal range with a very small edema (2e), score 3 a marked abnormality where the pericardial edema size were smaller than the head size (2f) and score 4 a major disorder with the size of the heart edema equal or even larger than the size of the head (2g).

### Experiment statistics

Three independent experiment replications, i.e. embryos collected from different clutches on different days, were performed for each dose group irradiated with plateau and mid-SOBP protons as well as 6 MV linac photons as reference. Individual replications comprise 96 embryos per dose group of 0 Gy, 5 Gy, 10 Gy, 15 Gy, 20 Gy and 30 Gy, i.e. a total of 576 embryos per experiment or 1728 embryos per radiation quality, respectively.

Survival rates were calculated by relating the number of surviving embryos at 1^st^, 2^nd^, 3^rd^ and 4^th^ day post irradiation to the number of embryos irradiated. For graphical representation of the average survival rates, determined as mean value of the three experiment replications, in dependence on dose the software Origin Lab 2017 (OriginLab Corporation, Northampton MA 01060, USA) was applied. Individual survival levels, i.e. effects at same doses but different radiation qualities, were compared by using the Log-Rank test with Bonferroni correction (GraphPad Prism Verion 7.04; GraphPad Software, La Jolla, USA). Statistical significant differences are indicated for p < 0.05. Since the embryonic survival curve could not be approximated by a classical linear-quadratic model, dose specific RBE values were calculated on basis of the average survival for the individual proton dose groups relative to the photon result. RBE uncertainties were derived by Gaussian error propagation using the corresponding standard deviations of the average survival rates.

The observed morphological abnormalities were quantified relating the number of malformed living embryos to the total number of living embryos at the corresponding day post irradiation. In doing so, the two abnormalities were considered individually, whereas the parallel appearance of both types of malformations was also recorded. Individual malformation rates at corresponding dose levels for proton and photon treatment were compared by using t-test applying a significance level of p < 0.05. Like for the embryonic survival, RBE values were calculated by relating the average malformation (pericardial edema or spinal curvature) rates obtained for certain proton and photon dose groups, respectively. Uncertainties of the RBE were again derived by Gaussian error propagation on basis of the standard deviations of the average malformation rates.

Qualitatively, mean scoring values (*SV*_*Mean*_) describing the average damage induced by one radiation quality were calculated on basis of the scored malformations on the 3^rd^ and 4^th^ day post irradiation:

$${SV}_{Mean}=\frac{\left(4*N_{score\;4}+3*N_{score\;3}+2*N_{score\;2}+N_{score\;1}\right)}{N_{living\;embryos}}$$

with N… representing the number of embryos scored for the respective category. As for the survival rates, the Origin Lab 2017 software was used to generate the graphical representation of time dependent occurrence of malformations and corresponding *SV*_*Mean*_.

## Results

### General outcome

Unlike experimentation in an animal laboratory, the study of radiobiological effects at accelerators is most often associated with environmental conditions that differ from the normal well-defined and stable conditions. In the present experiment campaign the ambient temperature come under the optimum of 28 °C, required for optimal development of zebrafish embryos, whenever the embryos were prepared, observed or irradiated. Room temperatures of 22.7–23.8 °C and of 22.0–23.0 °C were measured in the irradiation rooms and in the preparation laboratory, respectively. The water temperature in the phantom varied in the range of 21.7–23.5 °C. However, as notified by the low number of dead embryos directly after irradiation, the whole procedure, including non-optimal temperatures, transportation, positioning and irradiation, as well as the non-sterile conditions at the accelerators did not lead to significant embryo loss.

### Survival analysis

**Fig 3:**
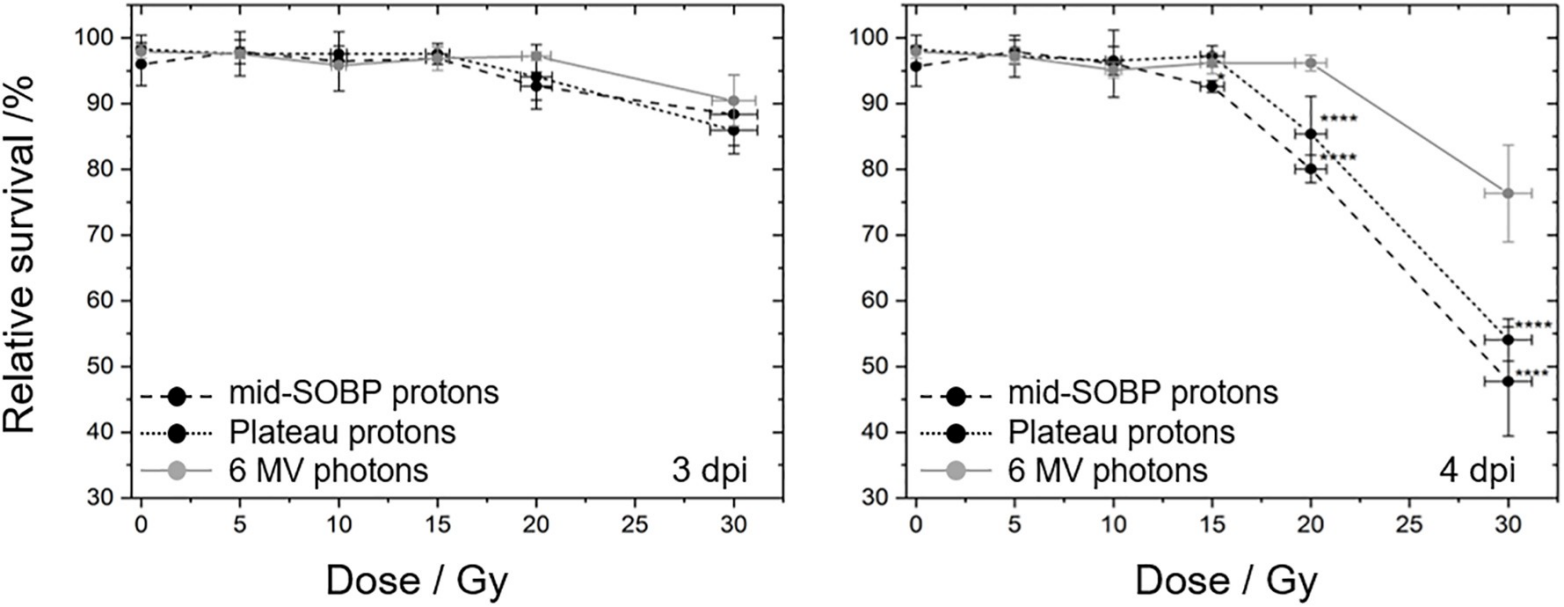
Radiation quality and dose dependent zebrafish survival curves. Zebrafish embryo survival rates at 3^rd^ (left) and 4^th^ (right) dpi with plateau (black, dotted) and mid-SOBP (black, dashed) protons relative to the survival after 6 MV photon reference irradiation (grey, solid). Error bars represent the standard deviation of three independent experiment replications (S1 Table); statistical significant differences to the photon reference are marked by * (p<0.05) and **** (p<0.0001).

The number of surviving embryos was recorded daily (S1 Table) starting with the first day after the treatment for four subsequent days. Dose dependent survival curves (Fig 3) were obtained correlating the number of living embryos of the respective day to the number of embryos irradiated. Thereby, the application of similar observation periods was intended for all experimental groups. However, due to different irradiation times for protons in the morning and photons in the evening, a time gap was introduced so that the observation at the 3^rd^ day was at an average of 2.96 dpi (range 89–99 hpf) and of 3.13 dpi (range of 93–103 hpf) and at the final day at 3.73 dpi (range of 111–118 hpf) and at 3.86 dpi (range of 114–119 hpf) for photons and protons, respectively. Regarding the survival curves (Fig 3) the introduced time gap results in a slight overexpression of the difference between protons and photons. But, as the detailed time dependence of the different endpoints between the 3^rd^ and the 4^th^ day post irradiation is not known, this time gap was not considered in the analysis.

In general, no significant impact of irradiation on embryonic survival was observed at the first two days post irradiation for all doses and at all days post irradiation for doses lower than 15 Gy. Beginning with the 3^rd^ dpi an influence on embryo survival was detected for proton doses higher than 10 Gy for SOBP and 15 Gy for plateau irradiation with significantly reduced survival levels at 4 dpi relative to the photon reference. This is also reflected by the LD_50_, the lethal dose required to kill 50% of the embryos, which was about 30 Gy for SOBP protons and slightly higher for plateau protons, whereas for photons considerably higher doses are required. RBE values for SOBP and plateau protons (Table 2) were calculated on basis of the survival levels measured at 4 dpi (S1 Table).

**Table 2:** RBE values obtained for the two proton radiation qualities.

	mid-SOBP	plateau
RBE_30Gy_± se	1.60 ± 0.32	1.41 ± 0.08
RBE_20Gy_± se	1.20 ± 0.04	1.13 ± 0.08

Dose dependent RBE values (± standard error) calculated on basis of the obtained survival rates 4 dpi with protons of the mid-SOBP and the entrance plateau relative to 6 MV photons.

### Malformations

**Fig 4:**
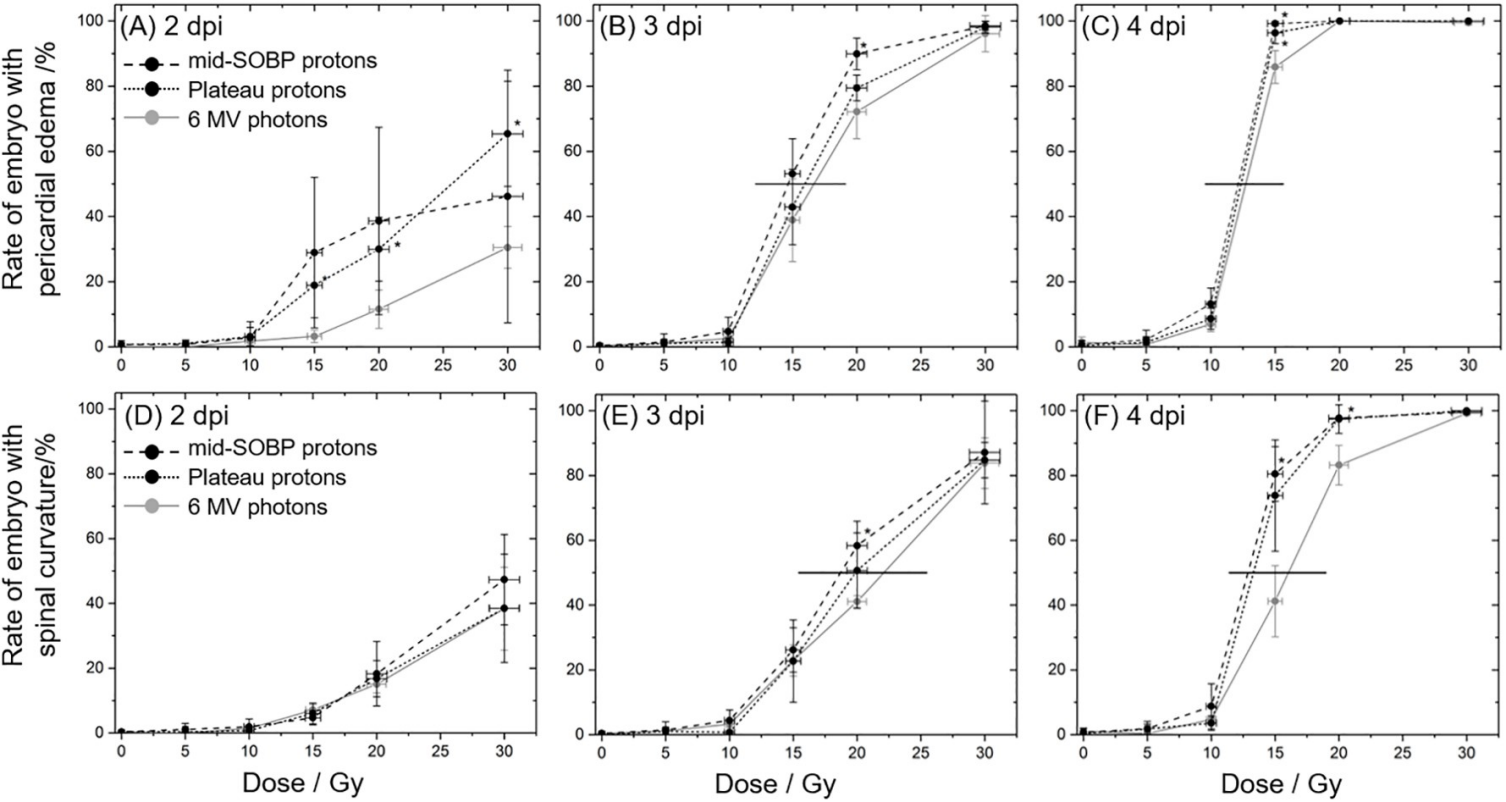
Radiation quality and dose dependent malformation rates. Time and dose dependent development of pericardial edema (A-C) and spine curvature (D-F) for zebrafish embryo irradiation with 6 MV photons (grey), proton plateau (black, dotted) and mid-SOBP position (black, dashed). Error bars represent the standard deviation of three independent experiment; statistical significant difference to the photon reference is indicated by * (p<0.05). Horizontal lines in the plots at 3^rd^ and 4^th^ dpi are shown to illustrate the differences in dose to induce 50% effect rate.

The occurrence of pericardial edema and spine deformations was investigated in parallel to embryonic mortality on each dpi. At the 1^st^ dpi, there were just a few malformations which appeared for high dose (> 20 Gy) treatments. In the following, the number of malformations rises from day to day, but again, doses below 15 Gy were less efficient in inducing malformations within 4 dpi (Fig 4, S2 Table). In general, observations at 1^st^ dpi were complicated by a high proportion of embryos in chorion, which might mask some malformations. At 2^nd^ dpi the majority of embryos were hatched, with hatching rates of 98–100% for the proton and of 86–90% for the photon treatments, respectively (S3 Table). At 3^rd^ and 4^th^ dpi almost 100% of the embryos were hatched and the spine as well as the pericardial region were clearly visible. The temporal progression of the two types of malformations under investigation is summarized in Fig 4 and it shows that the number of embryos with pericardial edema, as one of the acute reactions after irradiation, rises faster than the number of embryos with spine bending. Moreover, almost all embryos exhibiting a curved spine also suffered from pericardial edema (not shown), which also indicates the deterministic inflammation effect.

With respect to the different radiation qualities significant differences in the rates of pericardial edema between proton and photon treatment were predominantly found at the 2^nd^ and 3^rd^ dpi and for doses of 15 Gy and 20 Gy (Fig 4A–4C). At the 4^th^ dpi almost 100% of the embryos treated with doses higher than 15 Gy exhibited a pericardial edema and a significant difference was just revealed for the 15 Gy mid-SOBP irradiations. However, it should be mentioned, that the statistical variances obtained for the average malformation rates hampers the determination of significant differences between proton and photon irradiation. These variances might arise from systematic errors induced by the experiment, e.g., the time shift between the samples, or from differences in the timing of the inflammation process between individual embryos.

Comparing the malformation frequencies Fig 4 shows that the doses required to induce an unnatural curvature of the spine are higher than for the induction of pericardial edema. At 4 dpi almost 100% of the larvae treated with doses higher than 15 Gy exhibit an edema (Fig 4C), whereas a more differentiated and not so steep dose depending response was observed for spine bending (Fig 4D–4F) where a dose of 30 Gy is required to cause spinal curvature of almost all embryos.

Moreover, comparing the doses required to induce malformations in 50% of the embryos small temporal differences are revealed. For the induction of pericardial edema a dose distinction of 12.9 Gy for proton and 16.0 Gy for photon treatment was found at the 3^rd^ dpi, one day later a radiation quality independent dose of 12.5 Gy was measured (Fig 4C and 4F). For comparison, distinct dose levels of 18.6–22.1 Gy at the 3^rd^ dpi and of 14.7–16.7 Gy at the 4^th^ dpi were measured for spine deformations in 50% of the embryos after proton relative to photon treatment. For lower doses only a slight induction of malformations was found. On basis of the spinal curvature rates observed 4 dpi with 20 Gy protons RBE values of 1.25 ± 0.16 and of 1.10 ± 0.14 were calculated for the exposure in mid-SOBP and entrance plateau region relative to MV photons, respectively. For 30 Gy doses the rates of different radiation qualities are not distinguishable at all. The comprehensive occurrence of pericardial edema in all embryos at 4^th^ dpi makes the calculation of RBE pointless. At 3^rd^ dpi a proton quality independent RBE of 1.17 ± 0.10 could be derived for 20 Gy treatments.

**Fig 5:**
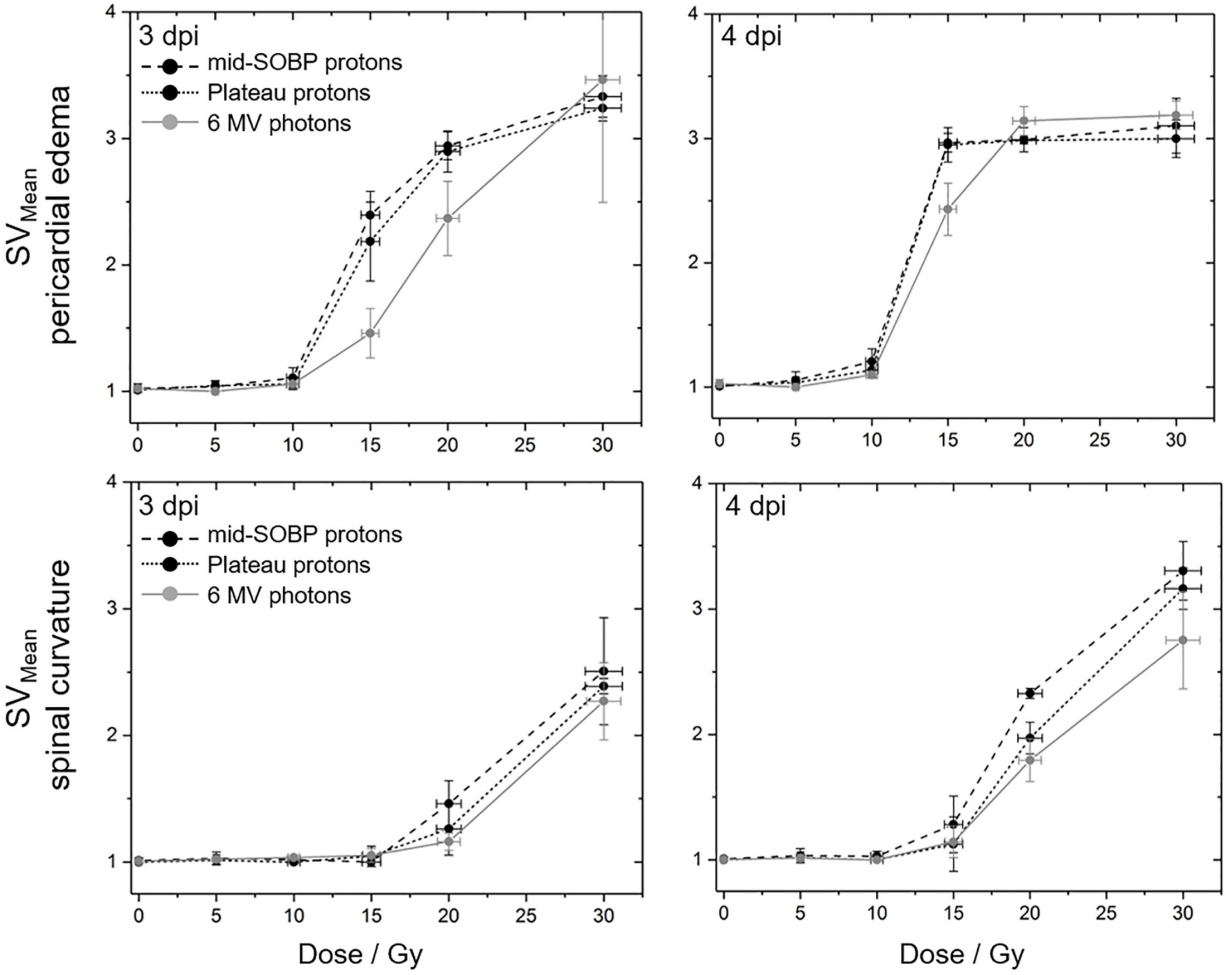
Qualitative evaluation of observed embryonic malformations. Mean scoring values describing the severity of the induced pericardial edema (upper row) and spine curvatures (lower row) at the 3^rd^ (left) and 4^th^ (right) dpi with mid-SOBP (black, dashed) or plateau (black, dotted) protons and 6 MV photons (grey). Error bars represent the standard error of three independent experiments; corresponding data are given in S4 Table.

The qualitative assessment of the severity of the induced malformations at 3^rd^ and 4^th^ dpi (Fig 5, data in S4 Table) reveal again that radiation quality alone does not result in significant differences with a trend towards less severe damage only transitionally observed 3 dpi with photons. Within the observation period of four days post irradiation sigmoidal dose response curves became manifest for the pericardial edema as one example of acute radiation damage. By contrast, for spine curvature, a linear dose dependent increase of severity up to the maximum tested dose of 30 Gy was observed at 4^th^ dpi probably caused by extensive cell death in the spine for doses above 10 Gy. However, saturation was also expected for higher doses or longer follow up times.

## Discussion

Zebrafish (*Dario rerio*) embryos are frequently used to assess different aspects of ionizing radiation, like radio-adaptive responses [25] and drug dependent radioprotection [16,26]. Moreover, the zebrafish embryo also represents a robust and simple alternative *in vivo* model for the assessment of radiation effects [13,15–17]. In this context, the present work aimed the establishment of the zebrafish embryo as model organism for the comparison of different radiation qualities and the determination of RBE, comparing clinical proton and photon beams. The radiobiological effects could be investigated with high spatial resolution due to their small size of about 0.5–1 mm, whereby in the present study the complexity of the experiment was reduced to the comparison of two proton beam depth positions in order to focus on the establishment of setup, workflow and endpoints.

As a by-product, the general applicability and robustness of this model under unfavorable conditions, i.e. non-sterile experimental rooms with less-than-ideal temperatures as well as transport and irradiation in a confined space, was verified by the very low number of deceased embryos throughout all experiments in the control groups and in all groups observed directly after irradiation. Moreover, the staging and slowing down of embryo development in accordance to Kimmel et al. [20] worked quite well, allowing the treatment of similarly developed embryos despite the shift in experiment time.

Consistent with the observation of Freeman et al. [25] the hatching rates observed in this study after irradiation during pharyngula period (S3 Table) show no significant dependence on radiation quality and dose. Hatching rates of 98–100% found at 2 dpi with protons are comparable to the control groups and to the level of > 95% found after ^60^Co gamma-irradiation [25]. The slightly reduced hatching rates of 86–90%, which were noticed at the second day after photon treatment could either be explained by the photon treatment itself or be a consequence of the delayed embryo development. Generally, most of the literature also shows a rather unaffected hatching rate after radiation treatment, which indicates that zebrafish embryos at pharyngula stage are quite radioresistant compared to those irradiated at earlier developmental stages e.g., [16, 26–28]. Few exceptions were published by Hu et al. [27] and Li et al. [29] who observed lower hatching rates two days after irradiation of 24 hpf embryos with 1 Gy of ^137^Cs photons [27] and 12 Gy of 8 MeV protons [29].

In accordance to the unaffected hatching process the survival of zebrafish embryos irradiated 24 hpf was also not significantly reduced by doses up to 15 Gy of MV photons and protons of the entrance plateau, and by up to 10 Gy of mid-SOBP protons (Fig 3), respectively. At higher doses the number of surviving embryos significantly declines, for proton treatment more efficient than for photons, whereas for protons the delivery of 30 Gy resulted in a 50% survival rate (LD_50_) already at the 4^th^ dpi. The dose threshold of 15 Gy for embryonic mortality observed at 4 dpi was also seen by others [16,26], whereas for lower energy protons of 8 MeV higher mortality rates were already revealed 4 days after treatment of 24 hpf wildtype embryos with a dose of 6 Gy [29]. Although the manuscript of Li et al. [29] does unfortunately not provide detailed information about beam delivery and dosimetry their findings confirm the higher biological efficiency of low energy protons at the Bragg peak and the distal-end of a therapeutic proton beam. Since 8 MeV protons have a range of about 0.8 mm in water [30] the zebrafish embryos in the experiment of Li et al. [29] are predominantly treated with high LET radiation which correlates to a significantly increased RBE. By contrast, the LET increase between entrance plateau and mid-SOBP position of a clinical 150 MeV proton beam [1] is not high enough to induce significant biological differences in embryos irradiated at the two positions in the present work.

Besides proton energy and LET, the age of the embryo at irradiation and the overall observation time clearly influence the mortality rate and consequently, the LD_50_. Regarding the latter, longer observation times, like for example the 7 dpi applied by Szabo et al. [16] after 20 Gy MV photon exposures, are required to notice the respective reduction in survival to 50% of the embryos. On the other side, embryos at earlier developmental stages than the 24 hpf used in the present work are more affected by radiation as shown by a mortality rate of 73.3% after 10 Gy ^60^Co gamma-irradiation of embryos at 3 hpf [28]. One potential explanation of the radioresistance of zebrafish embryo during pharyngula stage was provided by Hu et al. [27], who observed higher levels of proteins required for the defense against reactive oxygen species in 24 hpf compared to 6 hpf embryos.

In addition to the survival rates, the analysis of morphological abnormalities revealed that doses higher than 10 Gy are also required to trigger the appearance of pericardial edema and spinal curvature within the follow up time of four days. Similar threshold doses of about 10 Gy were also observed at 4 dpi of embryos treated during pharyngula stage with ^60^Co γ-rays [24] and MV photons [16]. Again, protons of 8 MeV are more efficient and doses of 3 Gy are already sufficient to significantly alter the normal development of 24 hpf embryos within the same observation period [29].

The slightly different timing between the two types of abnormalities observed in the present experiments could most likely be explained by their different genesis. The very fast and pervasive appearance of pericardial edema (cf. Fig 4) correlates with its emergence as acute inflammation reaction induced by cytokines released early after irradiation [31]. Consistently, the heart rate as a measure of physiological stress after radiation was found to decrease in a dose dependent manner after ^60^Co photon [25] or 8 MeV proton irradiation [29]. In contrast, the spinal malformations were probably caused by apoptosis of neural cells in the developing spinal cord [32], which needs some time to result in an observable abnormality after irradiation. The different genesis of the respective abnormalities might also be the reason for the small dose difference of 2.5 Gy found in the present work for the dose level required to induce the two types of abnormalities in 50% of the embryos within four days after irradiation.

Concerning the different radiation qualities, there are few measuring points at the 3^rd^ dpi and 4^th^ dpi (Fig 4) where significant differences were observable for the number of embryos with pericardial edema and with spine curvature, respectively. Also, the analysis of the severity of the induced damage on behalf of scoring values did not reveal a significant difference between radiation qualities, but show again the trend in correlation to the LET values and confirming the influence of time on the genesis of the two abnormalities. The differing curve shapes (Fig 5) verify the fast appearance of edema in almost all embryos, whereas the observed saturation level might indicate a tolerable limit. For the development of spinal curvature such a saturation level was not observed within the observation time but could probably be achieved by higher doses or by an extended observation time. Furthermore, the steep dose effect curve could be refined by additional doses between 10 Gy and 15 Gy.

One of the aims of the present study, besides the establishment of the experimental setup and reliable endpoints, was the derivation of RBE values for the two proton radiation qualities relative to the clinical MV photon beam. This has so far not been realized with aquatic animals. Concerning the embryonic survival, RBE values of 1.13 ± 0.08 and of 1.20 ± 0.04 were obtained at 4 dpi with 20 Gy of plateau and mid-SOBP protons, respectively. These values are in accordance with RBE values in the range of 0.96–1.13 and of 1.0–1.2 found in previous *in vivo* studies for treatments in the entrance plateau and mid-SOBP position [9, 33, 34], respectively. The increased RBE of 1.41 ± 0.08 and of 1.60 ± 0.32 for 30 Gy exposures at the respective positions rather matched RBE values found in cellular survival studies at the distal end of the proton path [3,5,6], whereby the achievement of the LD_50_ at this dose point might also have an influence on the validity of the derived RBE.

The few proton studies performed so far with zebrafish embryos used low energy protons, i.e. with a significantly higher LET than that delivered in the mid-SOBP in the present work, and aimed on the investigation of anti-angiogenic effects [35] or modifications of gene expression profiles [29] by proton treatment. Another point that should be considered is the general comparability of RBE data, since the biological effect depends not only on radiation quality, but also on species, dose fractionation, tissue or cell type, endpoint, timing etc. In this context, a comparison of RBE obtained on basis of zebrafish embryonic survival rates to other *in vivo* results, which are most often based on measurements of acute or late effects of a single organ in rodents (e.g. [8–10]), is critical. More detailed analysis of individual organs of zebrafish, like Urano et al. [33] did for mice, might help to better understand the proton induced effects and to resolve a meaningful difference between for example the pericardial and the spinal cord damage. In this context, the attempt to derive RBE values on basis of the induced malformation found at 4 dpi was feasible just for the spinal curvature, since pericardial edema as acute reaction are observed in all embryos. For the rates of spinal curvature RBE values of 1.25 ± 0.16 and of 1.10 ± 0.14 were revealed after treatment in mid-SOBP and entrance plateau position, which are comparable to those obtained on the basis of the embryonic survival rates and to RBE values determined by other *in vivo* models.

In conclusion, the present work validates the general applicability of zebrafish embryo as an alternative small vertebrate model for testing and assessing different radiation qualities, even under non-animal laboratory conditions. Qualitative endpoints like survival and malformations can be used to describe the general impact of radiation on the whole organism, whereas more detailed endpoints are required to resolve radiation damage to individual organs or on basis of molecular pathways. The parameters required for such investigations on radiation and biological effect relationships for the different endpoints are narrowed in the present work, e.g., more distinct dose dependencies of the malformation rates at the 3^rd^ dpi, and the obtained results warrant for further research on endpoint refinements. Perhaps, the application of other structured and comprehensive methods might help to resolve the underlying mechanisms of the indicated threshold dose for embryonic survival and the incidence of morphological abnormalities, respectively. A prolongation of the observation time is desirable and might result in more differential outcomes with respect to dependencies on dose and radiation quality. The potential application of zebrafish embryos for spatially resolved RBE measurements along the proton depth dose distribution seems to be conceivable.

## Supporting information

S1 Table Embryonic survival data obtained after irradiation with plateau and mid-SOBP protons as well as with 6 MV photon reference. Data from individual experiment replications and corresponding mean values and standard deviations, required for graphical representation and RBE calculation, are given.
(XLSX)

S2 Table Malformation rates measured daily post irradiation for the different radiation qualities. Absolute numbers of pericardial edema and spinal curvature were correlated to the number of surviving embryos. Results of individual experiment replications were given together with the derived mean values and standard deviations.
(XLSX)

S3 Table Hatching rates recorded daily after irradiation with plateau and mid-SOBP protons as well as 6 MV photon reference. (XLSX)

S4 Table Results of the quantitative assessment of the induced malformations. Mean scoring values in dependence on dose and time post irradiation were given for pericardial edema and spinal curvature.
(XLSX)

S1 Fig Typical GafChromic EBT3 film used to assess dose homogeneity. The exemplary film was irradiated behind a 96 well plate at the position of the entrance plateau and scanned two days after irradiation. Applying the corresponding calibration curve the film darkening was translated into radiation dose with high spatial resolution. Dose homogeneity was assessed by evaluating the doses within the zebrafish containing wells (dark orange circles) within the irradiated field marked by the red square.
(TIF)
