# Supplementary figures and images for: Radiobiological effects and proton RBE determined by wildtype zebrafish embryos
Source: PLoS One. 2018 Nov 8;13(11):e0206879. doi: 10.1371/journal.pone.0206879 (PMC6224071; doi:10.1371/journal.pone.0206879)

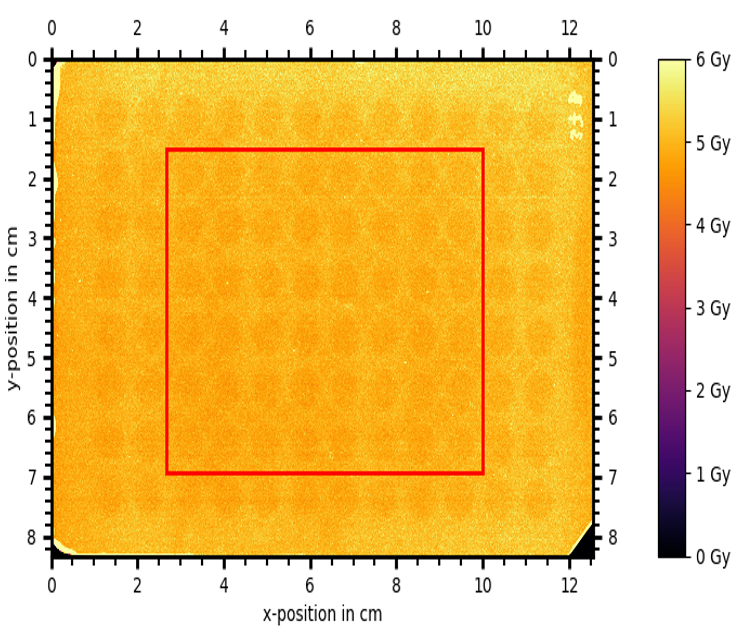

Supplement: S1 Fig — The exemplary film was irradiated behind a 96 well plate at the position of the entrance plateau and scanned two days after irradiation. Applying the corresponding calibration curve the film darkening was translated into radiation dose with high spatial resolution. Dose homogeneity was assessed by evaluating the doses within the zebrafish containing wells (dark orange circles) within the irradiated field marked by the red square. (TIF) [file pone.0206879.s005.tif]
